# Probiotics as a treatment for prenatal maternal anxiety and depression: a double-blind randomized pilot trial

**DOI:** 10.1038/s41598-021-81204-9

**Published:** 2021-02-04

**Authors:** Pamela D. Browne, Antoinette C. Bolte, Isolde Besseling-van der Vaart, Eric Claassen, Carolina de Weerth

**Affiliations:** 1 Department of Cognitive Neuroscience, Donders Institute for Brain, Cognition and Behaviour, Radboud University Medical Center, Kapittelweg 29, 6525 EN Nijmegen, The Netherlands; 2 Faculty of Earth and Life Sciences, Athena Institute, VU University, De Boelelaan 1085, 1081 HV Amsterdam, The Netherlands; 3 Department of Obstetrics and Gynecology, Radboud University Medical Center, Geert Grooteplein Zuid 10, 6525 GA Nijmegen, The Netherlands; 4 Winclove Probiotics BV, Amsterdam, The Netherlands

**Keywords:** Psychology, Gastroenterology, Medical research, Signs and symptoms

## Abstract

Probiotic use may be an efficacious treatment option to effectively manage symptoms of prenatal maternal anxiety and depression. Our primary aim was to test feasibility and acceptability for a probiotic randomized controlled trial (RCT) in pregnant women with pre-existing symptoms. This double-blind pilot RCT included 40 pregnant women with low-risk pregnancies and elevated depressive symptoms and/or anxiety. Once daily, participants orally consumed a probiotic (Ecologic Barrier) or a placebo, from 26 to 30 weeks gestation until delivery. A priori key progression criteria for primary outcomes were determined to decide whether or not a full RCT was feasible and acceptable. Secondary outcomes included depressive symptoms, anxiety, stress, and maternal bonding to offspring. In 19 months, 1573 women were screened; following screening, 155 women (10%) were invited for participation, of whom 135 (87%) received study information, and 40 women (30%) were included. Four out of six a priori determined criteria for success on feasibility and acceptability were met. After 8 weeks of intervention, there was no significant difference between the probiotic and placebo groups for secondary outcomes. The pilot trial was feasible and acceptable, but hampered by recruitment method and study design. Secondary endpoints did not reveal differences between the groups for improving maternal mood.

## Introduction

Prenatal maternal depression and anxiety are risk factors for adverse outcomes in mothers and children^1,2^. Simple treatments that effectively manage these symptoms in most women are greatly lacking^3^. This emphasizes the value of investigating potential efficacious adjuvant treatment options for pregnant women with these symptoms. Increased depressive symptoms and heightened anxiety are suggested to have a complex and multi-factorial etiology with multiple factors interacting and contributing to the development of symptoms^4^. These factors include personal resources, predisposing demographic factors, antenatal stressors^5,6^, and socio-cultural beliefs^7^. Other factors include physiological and epigenetic factors^8^, and possibly gut microbiota^9,10^.

The gut microbiota is suggested to communicate with the brain through nervous, endocrine, and immune signaling mechanisms, also referred to as the ‘microbiota-gut-brain-axis’^11,12^. Via this axis, alterations in the gut microbiota composition may contribute to the development of depressive and anxiety symptomatology^13,14^. Probiotics can affect gut microbiota and hence may reduce depressive symptoms and/or heightened anxiety in people with pre-existing symptoms^9,15,16^. Probiotics are defined as “live organisms that, when consumed in adequate amounts, confer a health benefit to the host”^17^. A randomized double-blind placebo-controlled trial (RCT) in pregnant women found that probiotics ingested during the perinatal period reduced depressive and anxiety symptoms during the postnatal period^10^. To date, no RCTs exist that investigate the possible efficacy of probiotics to improve maternal mood prenatally.

In psychology and medicine, RCTs are considered “gold standard” to evaluate therapeutic efficacy of new treatments^18,19^. Success of an RCT is largely determined by adequate recruitment of participants and compliance with the study protocol during a RCT^20,21^. Inadequate recruitment and non-compliance have negative consequences for participants, investigators, and in some cases public funds, and should therefore be prevented^22,23^. Pilot trials provide an excellent method to avoid these issues by assessing *feasibility* (i.e., whether potential participants would be willing to participate in the trial) and *acceptability* (i.e., whether the design of the trial is acceptable to participants) of a full RCT^24^.

The primary aim of this pilot RCT was to evaluate the *feasibility* in terms of recruitment and retention, and *acceptability* in terms of compliance and participants’ experiences of daily probiotic intake and the collection of data. The secondary aim of the present study was to exploratorily investigate the potential effects of probiotic intake, compared to placebo, on depressive symptoms, anxiety, stress (from here on named ‘psychosocial distress’), and by potentially affecting mood, maternal bonding to the offspring^25,26^.

## Methods

### Pilot RCT

The current pilot trial evaluated the *feasibility* and *acceptability* of a full RCT (a prenatal intervention on perinatal outcomes). Hence, the study design of the current pilot RCT reflected the design of the potential full RCT. The design reflects an integrative research approach combining both validated questionnaires and biological measures. This approach has been shown successful in many of our previous studies^27–31^ and previous probiotic trials^9^. A description of the ethics, participants, in- and exclusion criteria, trial design, randomization*,* blinding, treatment allocation and sample size of the pilot trial are provided below. Full details about the procedures, methods and outcome measures are described in detail in a separate paper^32^.

#### Ethics

The Medical Ethics Committee (METC) of the Radboud University Medical Center in Nijmegen, the Netherlands (NL57780.091.16) approved the study. Study procedures were conducted in accordance with the Helsinki Declaration of 1975 as revised in 1983. The trial was registered under trial number NTR6219 on 28/02/2017 (Netherlands Trial Register).

#### Participants

Forty healthy pregnant women (≥ 18 years) with at least mild depressive symptoms and/or anxiety in the late second/third trimester of an uncomplicated pregnancy (≥ 26 and ≤ 30 weeks gestation) participated in this study.

#### Inclusion and exclusion criteria

Inclusion criteria were: (1) elevated levels of depressive symptoms (EPDS ≥ 10) and/or anxiety (STAI-S ≥ 40), (2) start daily probiotic/placebo product intake between 26 and 30 weeks gestational age and continue until delivery. Exclusion criteria include: (1) multiple pregnancy, (2) high suicidal risk according to the suicidality subscale score on the MINI International Neuropsychiatric Interview, (3) illegal drug use, (4) psychiatric history of psychoses or bipolar disorder, (5) inflammatory bowel disease, (6) other autoimmune disorders and/or treatment with immunosuppressive therapy, (7) known pre-existing diabetes mellitus, hyperemesis gravidarum, hypertensive disorder, liver and/or renal disease, (8) malignancy and/or treatment with radiation or chemotherapy, (9) history of major gastro-intestinal surgery, (10) allergy or hypersensitivity to any ingredients in the Ecologic Barrier/placebo product, (11) history of using Ecologic Barrier, (12) presently using food containing probiotics (probiotic intake needed to stop at least 2 weeks prior to the start of the probiotic/placebo product intake), (13) not speaking and/or writing Dutch^32^.

#### Trial design

From 26 to 30 weeks gestation until delivery, participants once daily orally consumed a probiotic multispecies mixture (Ecologic Barrier; 2.5 × 10^9^ CFU/g; daily dosage 2 g; *Bifidobacterium bifidum* W23, *Bifidobacterium lactis* W51, *Bifidobacterium lactis* W52, *Lactobacillus acidophilus* W37, *Lactobacillus brevis* W63, *Lactobacillus casei* W56, *Lactobacillus salivarius* W24, *Lactococcus lactis* W19 and *Lactococcus lactis* W58) or a placebo. The probiotic product and placebo were indistinguishable regarding color, taste, and smell (Winclove Probiotics BV, Amsterdam, the Netherlands). Given that a potential full RCT would not investigate probiotics as a single management option for psychosocial distress, but the potential effectiveness in addition to participants’ current treatment, the use of any kind of co-medication or therapy was allowed.

To assess the potential effect of prenatal probiotic intake compared to placebo on perinatal maternal psychosocial distress women filled in questionnaires at baseline (time point 0 (t0)) and 8 weeks after starting probiotic/placebo intake (time point 1 (t1)). Additionally, to assess the potential long-term effects of prenatal maternal probiotic intake, maternal mood and maternal bonding to offspring were assessed 4 weeks post-partum (time point 2 (t2)).

#### Randomization, blinding, and treatment allocation

Forty women were randomly allocated to either the probiotic group or placebo group by a computer random number algorithm (1:1 allocation ratio), using blocks of four. Research personnel, who provided the probiotic or placebo and collected data, and participants were blinded to the treatment assignment and uninformed about the randomization sequence. Outcome assessors were also blinded to the treatment assignment. Further details on the intervention, intake, randomization and probiotic/placebo delivery procedure can be found in the protocol manuscript^32^.

#### Sample size

A sample size of 40 participants (~ 27% of a full trial) was deemed appropriate to inform about the primary outcomes. Based on a previous study by Steenbergen et al. (2015), which investigated the effect of the same probiotic product on mood in a non-pregnant healthy population, this sample size was also regarded sufficient to exploratorily assess the potential effects of probiotics^33^.

### Primary outcomes of the pilot RCT

The primary outcomes of this pilot RCT were the feasibility and the acceptability. Feasibility was assessed in terms of recruitment (i.e., the proportion of pregnant women with psychosocial distress accepting the invitation to participate in the pilot trial and reasons for (non) participation) and retention (i.e., the number of women completing the study from enrolment to follow-up). Acceptability was measured in terms of compliance (i.e., the proportion of participants adhering to the intervention schedule and the collection of data) and participants’ experiences during the pilot trial. Based on these primary outcomes, six a priori key progression criteria were established, see Table 1. Not meeting one or more key progression criteria indicates that the study protocol needs to be revised before conducting a full RCT.

**Table 1:** Primary quantitative outcomes, outcome measures and time point for each outcome (i.e., baseline (t0), 8 weeks after probiotic/placebo intake (t1) and 4 weeks post-partum (t2), (key) progression criteria for a full RCT, and (key) progression criteria that were met (yes/no).

Primary quantitative outcomes	Outcome measure, time point for each outcome	Progression criteria for full RCT	Progression criteria met (yes/no)
A) Recruitment	1) The proportion of contacted potential participants meeting the inclusion criteria and who consent to participate in the pilot trial	**30 participants recruited in 6 months**	No (40 participants in 19 months)*
	2) The number of participants recruited at each recruitment site for the research database	No criteria set	–
	3) Reasons for (non) participation	No criteria set	–
B) Retention rate	1) The proportion of participants completing the study from enrolment (t0) to follow-up (t2)^1^	** ≥ 90% of participants**	Yes (98% of participants completed the pilot trial)*
C) Compliance	1) The proportion of participants taking ≥ 80% probiotic/placebo product between t0 and t1	**90% of participants**	Yes (90% of participants)*
	2) The proportion of participants filling in electronic questionnaires at all time points	**90% of participants**	Yes (100% of participants)*
	3) The proportion of participants filling in the cry diary at t2	90% of participants	No (85% of participants)
	4) The proportion of participants filling in evaluation form at t2	90% of participants	Yes (98% of participants)
	5) The proportion of participants collecting maternal vaginal samples at t0 and t1	90% of participants	No (83% of participants)
	6) The proportion of participants collecting maternal microbial samples at t0 and t1	90% of participants	No (85% of participants)
	7) The proportion of participants collecting infant microbial samples at t2a and t2b	90% of participants	No (83% of participants)
	8) The proportion of participants allowing collection of hair samples at t2	90% of participants	No (83% of participants)
D) Participants’ experiences	1) Level of acceptability of electronic questionnaires	No criteria set	-
	2) Level of acceptability collection of biological samples^2^	No criteria set	-
	3) Level of satisfaction (positive/negative aspect)	No criteria set	-
	4) Level of satisfaction (execution and organization)	No criteria set	-
	5) Level of acceptability of the pilot trial in practice (i.e., perceived burden)	**Mean score of ≤ 3**	No (Mean 4; SD 2.2)*
	6) Level of acceptability of the pilot trial in principle (future participation)	**Mean score of ≥ 8**	Yes (Mean 8; SD 1.7)*

Key progression criteria are bold.

^1^Full completion of the study defined as: the closing home visit has taken place at t2.

^2^Of the 34 participants who collected biological samples.

*Key progression criteria. If one of more key progression criteria is not met revisions to study protocol should be made prior to conducting a full RCT.

#### Recruitment

In order to recruit 40 participants, local advertisement to participate in an online questionnaire study on mood and experiences during pregnancy was provided at six sites in the Netherlands: two ultrasound centers, an academic hospital, a lactation consultant practice, and two mental health centers. Participants meeting the criteria for inclusion were invited to participate in the pilot RCT; they were included in the study after providing written informed consent. Additional information of the way participants were identified and consent was obtained can be found in the protocol publication^32^.

#### Retention

The proportion of participants completing the study from enrolment (t0) to follow-up (t2) was recorded on Case Report Forms (CRFs) throughout the study.

#### Compliance

Data on the proportion of participants taking ≥ 80% probiotic/placebo product was collected at t2 by the CI by counting the sachets that were left from the previous weeks.

#### Participants’ experiences

A self-created evaluation form was used to assess participants’ experiences during the pilot trial. The questionnaire included nine questions in total. Five open questions on experiences of filling in questionnaires during the pilot trial (i.e., length, number of questionnaires, electronic method), the collection of maternal/infant stool and vaginal samples (i.e., number of samples, explanation about the method of collection, potential difficulties in collecting samples), and the collection of the hair sample; and two open questions on the level of satisfaction (“Regarding the study in general, which aspects did you experience as most positive and negative?” “Do you have any general remarks regarding execution and organization of this study?”). Additionally, two closed questions on the level of satisfaction were included (i.e., acceptability of the pilot trial in principle and in practice). To rate the acceptability of the pilot trial in principle, participants indicated the likelihood of participating in a similar trial in the future (0 = ‘no chance of future participation’ to 10 = ‘very high chance of future participation’); for the acceptability of the pilot trial in practice participants rated on a scale of 0 to 10 the perceived burden of the trial (0 = ‘no burden’ to 10 = ‘very large burden’).

### Secondary outcomes of the pilot RCT

A brief description of all measurement tools used can be found below; an elaborate description, as well as a description of other measures not used in the analyses for the current manuscript, is provided in the protocol paper^32^.

#### Demographic characteristics

Participants filled out questionnaires on demographics, health behavior and lifestyle. Demographic variables were collected during the screening phase (time point -1 (t-1)).

#### Maternal depressive symptoms

##### Edinburgh Postnatal Depression Scale

Pre- and postnatal depressive symptoms were measured using the Edinburgh Postnatal Depression Scale (EPDS), which is a validated tool to screen for antenatal and postnatal depressive symptoms^34^. The EPDS consists of 10-items scored on a 4-point scale with a maximum score of 30. A sum score of 10 or more was considered to reflect the presence of at least mild depressive symptoms^35^.

##### Leiden Index of Depression Sensitivity-Revised

Negative thoughts associated with sad mood, as a predictor of depression, was measured with the validated Leiden Index of Depression Sensitivity-Revised (LEIDS-R)^36^. The questionnaire consists of 34 items for which participants indicate to what extent each statement applies to them on a 5-point Likert scale. A higher total score indicates more cognitive reactivity to sadness, which indicates a potential larger tendency to develop depression.

#### Maternal anxiety

##### Pregnancy-Related Anxiety Questionnaire-Revised (Pregnancy-related anxiety)

Pregnancy-specific anxiety was measured using the Pregnancy Related Anxiety Questionnaire-Revised (PRAQ-R), which is a validated 10-item tool scored on 5-point scale that screens for pregnancy-related anxiety in pregnant women^37^. Two subscales “fear of giving birth” and “fear of bearing a handicapped child” were used. Higher scores on these subscales indicate more pregnancy-related anxiety.

##### State-trait Anxiety Inventory (general anxiety)

The state version of the State-trait Anxiety Inventory (STAI-S), a validated self-report questionnaire for pregnant women, was used to measure general anxiety^38^. The STAI-S consists of 20 statements related to current feelings of anxiety, which are scored on a 4-point Likert scale (maximum score of 80). Higher scores indicate more general feelings of anxiety. A cut-off score of 40 has been shown to have high sensitivity and specificity among pregnant women in their third trimester of pregnancy to detect anxiety disorder^39^.

#### Maternal stress

##### Pregnancy Experience Scale (pregnancy-related daily hassles)

The validated Pregnancy Experience Scale (PES) was used to measure maternal appraisal of daily, pregnancy-specific hassles and uplifts^40^. The questionnaire consists of 43-items on which participants rated the degree to which these specific experiences constituted a hassle and uplift on two separate 5-point Likert scales. To calculate the total intensity score for hassles, a sum of scores for hassles was calculated and divided by the number of endorsed items for hassles. A similar calculation for total intensity score for uplifts was performed. A composite ratio score was computed relating hassles to uplifts (i.e., total intensity score for hassles divided by total intensity score for uplifts). A ratio larger than 1 indicate more hassles than uplifts (i.e., larger negative emotional valence towards pregnancy); a ratio smaller than 1 indicate more uplifts than hassles.

##### Everyday Problem List (general daily hassles)

The validated 49-item Dutch Everyday Problem List (APL) measured the occurrence and intensity of general daily hassles during pregnancy^41^. Participants indicated which specific daily hassles they had experienced and scored on a 4-point Likert scale how much each of them had bothered them. The total sum of the Likert scale points was divided by the total number of daily hassles reported. A higher value indicate more perceived stress due to daily hassles.

#### Mother to infant bonding

##### The Maternal Antenatal Attachment Scale

The validated Maternal Antenatal Attachment Scale (MAAS) assessed participant’s bonding with her fetus^42^. The MAAS consists of 19 items scored on a 5-point Likert scale. Higher total sum scores indicate higher preoccupation with the unborn child and higher quality bonding.

##### The Maternal Postnatal Attachment Scale

The Maternal Postnatal Attachment Scale (MPAS), a validated tool among pregnant women, was used to measure the strength of the mother to infant bonding^43^. The MPAS questionnaire consists of 19 items that are scored on a 5-point Likert scale. Higher total sum scores reflect higher quality of maternal to infant bonding.

### Potential confounders

Previous literature reported several variables that are associated to maternal mood^11,44^. These include sleep, stressful events, and the use of (non) pharmacological treatments (e.g. yoga-based interventions)^45^. Data on these variables were collected at baseline (t0) and 8 weeks after treatment (t1) to control for undesired differences between the treatment and placebo groups that may confound potential treatment effects.

#### Sleep

Sleep (i.e., quality and disturbances during the previous month) was measured using the validated Pittsburgh Sleep Quality Index (PSQI). The questionnaire consists of 19-items, which are scored on a 4-point Likert scale. A total sum score of > 5 indicates poor sleep quality^46^.

#### Stressful events

In the current absence of a validated questionnaire on stressful life events for pregnant women, we constructed a 7-item stressful event questionnaire. The items were based on previous literature on predictors of prenatal anxiety and depression^4,47^. In the questionnaire, participants listed whether they experienced a stressful event (yes/no) during the first eight weeks of probiotic intake. These items included: “sickness of oneself”, “sickness of partner”, “death of a close family member/friend”, “relational/marital problems”, “divorce/separation”, “financial problems”, “domestic violence”, “other” (See Supplementary Fig. 1). Items were summed up so that more reported stressful events were indicative for higher incidence of stressful life events during the probiotic/placebo product intake period.

#### (Non) Pharmacological treatments

Regarding the use of **(**non) pharmacological treatments, participants were asked whether they started to use new (non) pharmacological treatments during the intervention period (yes/no).

#### Side effects and serious adverse events

Side effects (“constipation”, “flatulence”, “nausea”, “diarrhea”, “other”) were recorded at baseline (t0) and 8 weeks after probiotic/placebo intake (t1). Serious adverse events (SAEs) were recorded throughout the study.

### Statistical analyses

#### Qualitative analysis for primary outcomes

Qualitative data were analyzed according to the principles of the Framework approach^48^. Two researchers (PD and HL; see Acknowledgements) independently developed an analytical framework by reading through all responses on the interview questions and by coding the transcripts separately. The researchers consequently compared the codes and once agreed to a set of codes, grouped them together to form themes and categories to establish an overarching analytical framework. The analytical framework was then used to index all transcripts using an Excel spreadsheet chart (Microsoft Excel, version 14.6.6, 2011).

#### Quantitative analyses for primary and secondary outcomes

Demographic characteristics of participants were analyzed using descriptive statistics as mean/median and percentages. Following the intention-to-treat principle, data of all participants were included in the statistical analyses. Mean/median and frequencies were compared using *t*-tests and Fisher’s exact test, respectively. For each questionnaire, mean total scores were initially compared between placebo and probiotic groups by two-sample independent *t*-tests to detect between-groups effects at baseline (t0), after 8 weeks of probiotic/placebo intake (t1) and 4 weeks post-partum (t2). Data were tested for equal variance and where data did not have equal variance, Welsh’s correction was used. To detect within-groups differences over time, repeated measures ANOVA were performed with time (t0, t1 and t2) as within-subject factor and group (placebo vs. probiotic) as between-subjects factor. Tukey HSD *post-hoc* test was used to identify significant differences in the means between pairs. A *p* value of < 0.05 was considered significant. All analyses were performed with the use of R software (R studio, version 1.1.463, 2018; www.r-project.org). Regarding the primary outcome on participants’ experiences, themes and categories were calculated as a percentage of the total number of participants reporting that specific theme or category (see *Qualitative analysis for primary outcomes* for qualitative analysis method).

## Results

### Preliminary analyses

The probiotic and placebo groups did not significantly differ with regard to maternal characteristics (t0; *M* = 27.8 weeks gestation, *SD* = 1.5) (independent t-test; Fisher’s exact test) (see Table 2). Additionally, there were no significant differences between probiotic and placebo groups for starting new (non) pharmacological treatments between pre-treatment (t0; *M* = 27.8 weeks gestation, *SD* = 1.5) and t1 (*M* = 35.8 weeks gestation, *SD* = 1.5) (Fisher’s exact test, *p* > . 05). Furthermore, no significant differences were found for the number of reported stressful life events during the first 8 weeks of probiotic intake for participants in the probiotic versus placebo group [F(1,38) = 0.176, *p* > 0.05]. There was a significant effect of time on sleep [F(1, 38): 8.72, *p* < 0.05], with sleep quality worsening over pregnancy for the group as a whole. However, no significant effects of time by group interaction were found [F(1,38) = 0.29, *p* > 0.05]. In conclusion, new (non) pharmacological treatments, stressful events, and quality of sleep were not regarded as confounding variables for differences between the groups in depressive symptoms, anxiety or stress, and therefore not included in the analyses as confounders.

**Table 2:** Pre-treatment demographic characteristics of women receiving probiotic or placebo product.

	Probiotic (N = 20)	Placebo (N = 20)
Age (years) [Mean, *SD*]	29.65 (3.9)	31.7 (4)
**Educational level [n (%)]**		
Lower secondary education	0 (0%)	2 (10%)
Lower secondary vocational education	4 (20%)	3 (15%)
Higher secondary education	1 (5%)	0 (0%)
Tertiary education	15 (75%)	15 (75%)
Number of previous pregnancies [median (*IGR*)]	1.5 (1.25)	2 (1)
**Marital status [n (%)]**		
Married/registered partnership	15 (75%)	13 (65%)
Unmarried with partner	5 (25%)	6 (30%)
Single	0 (0%)	1 (5%)
**Employment status [n (%)]**		
Employed	16 (80%)	19 (95%)
Unemployed	4 (20%)	1 (5%)
Ethnicity [n (%)]		
Dutch	20 (100%)	18 (90%)
Other	0 (0%)	2 (10%)
Weeks gestation at start of intervention [median (*IGR*)]	28 (2)	28 (2.25)
**Psychiatric diagnosis [n (%)]**		
Depression	1 (5%)	2 (10%)
Anxiety disorder	2 (10%)	2 (10%)
Borderline disorder	0 (%)	1 (5%)
Posttraumatic stress disorder	0 (0%)	1 (5%)
Pervasive Developmental Disorder Not Otherwise Specified (PDD-NOS)	1 (5%)	0 (0%)
**History of depression [n (%)]**^**1**^		
Prenatal depression	0 (0%)	1 (5%)
Postpartum depression	2 (20%)	6 (30%)
Non- pharmacological treatment(s) [n (%)]	9 (45%)	6 (30%)
Cognitive behavioral therapy	1 (5%)	2 (10%)
Psychotherapy	2 (10%)	0 (0%)
Rapid eye-movement therapy	1 (5%)	1 (5%)
Psychosocial support	4 (20%)	2 (10%)
Haptotherapy	1 (5%)	1 (5%)
Pharmacological treatment(s) for depression or anxiety [n (%)]	1 (5%)	3 (15%)
Food supplement(s) use [n (%)]^2^	6 (30%)	3 (15%)
Vitamin(s) use [n (%)]	16 (80%)	18 (90%)
Smoking [n (%)]	0 (0%)	2 (10%)
Alcohol [n (%)]	0 (0%)	0 (0%)
**Diet [n (%)]**		
Lactose free	0 (0%)	1 (5%)
Biological food	0 (0%)	1 (5%)
Gluten free	1 (5%)	0 (0%)
Vegetarian (no meat)	0 (5%)	2 (10%)
Vegan	0 (0%)	0 (0%)
**Baseline mood scores [mean (SD)]**		
Depressive symptoms^3^	12.8 (4.3)	12.5 (3.9)
Cognitive reactivity^4^	49.4 (10.4)	43.45 (10)
Pregnancy-related anxiety—fear handicapped child^5^	8.7 (3)	9.7 (4.5)
Pregnancy-related anxiety—fear of birth^6^	7.4 (2.9)	7.65 (3.2)
General anxiety^7^	47.75 (9.1)	47.55 (9.9)
General daily hassles (total score)^8^	0.71 (0.33)	0.71 (0.31)
Pregnancy-related daily hassles (uplifts)^9^	1.50 (0.43)	1.45 (0.41)
Pregnancy-related daily hassles (hassles)^9^	0.96 (0.4)	0.99 (0.34)
Mother to infant bonding^10^	66.8 (8.04)	65.5 (6.44)

^1^Out of 10 women in probiotic group and 13 women in placebo group with previous pregnancies.

^2^(Homeopathic) iron supplements, bee pollen etc.

^3^Edinburgh Postnatal Depression Scale (EPDS); scale: 0–30^34^.

^4^Leiden index of depression sensitivity-revised (LEIDS-R); scale 0–136^51^.

^5^Pregnancy Related Anxiety Questionnaire-Revised (PRAQ-R); scale 3–20^37^.

^6^Pregnancy Related Anxiety Questionnaire-Revised (PRAQ-R); scale 3–15^37^.

^7^State Trait Anxiety Inventory (STAI-S); scale: 20–80^38^.

^8^Everyday Problem Checklist (APL); (-1)–1^41^.

^9^Pregnancy Experience Scale (PES); scale 0–4^40^.

^10^Maternal Antenatal Attachment Scale (MAAS); scale 19–95^42^.

**Figure 1:**
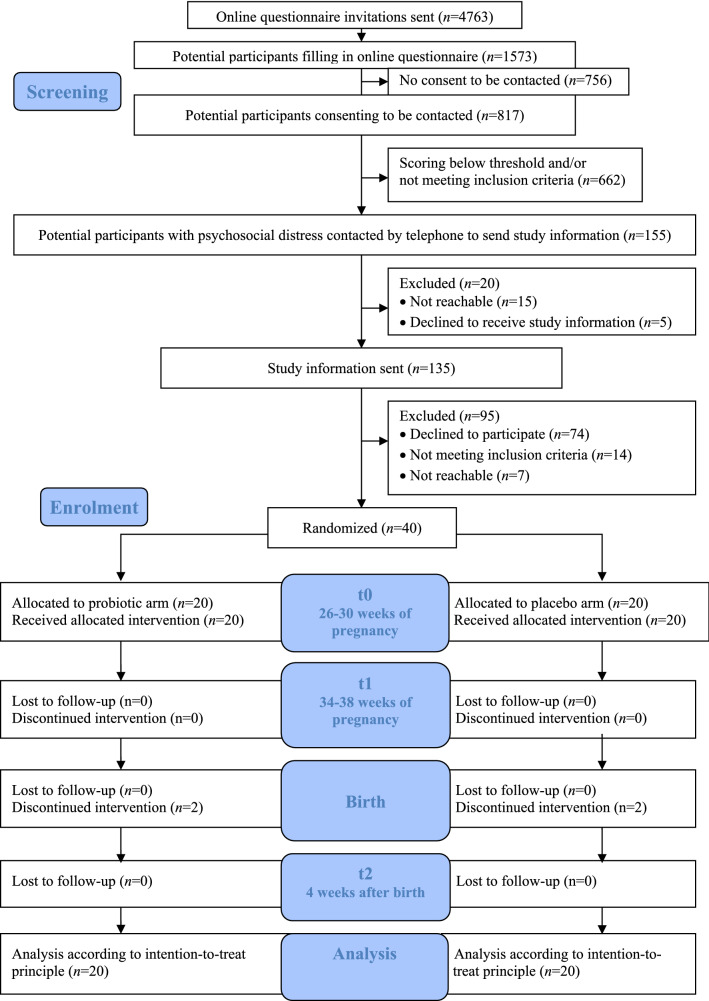
Participants’ flow through the study based on the Consolidated Standards of Reporting Trials (CONSORT) flow diagram. From the research database, participants were screened for eligibility and contacted by phone by the coordinating investigator whether they wanted to receive study information. Eligible participants who provided consent were randomized in either the probiotic or placebo group (t0). Measurements took place between baseline (t1) and 4 weeks post-partum (t2). Participants were recruited from March 2017 to September 2018.

### Primary outcomes

Four out of the six key progression criteria were met: 98% of participants completing the study (i.e., the closing home visit took place at t2 to finalize the study), 90% of participants taking ≥ 80% probiotic or placebo product for eight weeks straight, 100% of participants filling in online questionnaires at all time points, and acceptability of the pilot in principle (mean score of 8, *SD* = 1.7). The progression criterion for recruitment (40 participants recruited in 19 months) and the acceptability of the pilot in practice (mean score of 4, *SD* = 2.2) were not met (see Table 1).

#### Recruitment

Participants were recruited from March 2017 to September 2018 for the research database by local staff. During the first three months of recruitment (March–May 2017) it appeared that only 2 of the 14 potential participants consented to participate. In order to improve the recruitment rate, a second echo center (September 2017), an academic hospital (November 2017), a lactational practice (December 2017), and two mental healthcare centers (January 2018) were additionally added as recruitment sites.

Regarding the recruitment rate, of the 1573 potential participants filling in the online questionnaire, 817 of them consented to be included into the research database to be contacted for other research on psychosocial distress. Of the 817 women, 155 (19%) scored above the threshold for psychosocial distress and met the study inclusion criteria. Most of the 155 women agreed to receive study information (*n* = 135; 87% of the women contacted by phone) and finally 40 women agreed to participate in the pilot trial (30%) (see Fig. 1).

With respect to the number of participants recruited at each of the recruitment sites for the research database, Table 3 shows the recruitment numbers per recruitment site. As can be observed in the table, most participants were recruited through the echo centers. At echo center 1, a total of 4147 email invitations were sent to potential participants, of which 1088 women fully completed the questionnaire (response rate 26%). Regarding echo center 2, a total of 596 email invitations were sent and 441 women filled in the questionnaires (response rate 74%). At the academic hospital email addresses from potential participants were collected and a link of the online questionnaire was provided on flyers on site; a total of 20 email invitations were sent to women visiting the academic hospital. At the lactational practice and mental healthcare centers, only a link of the online questionnaire was provided on flyers and therefore the response rate could not be calculated*.*

**Table 3:** Recruitment method, recruitment period, number of potential participants who filled in the online questionnaire, average number of filled in online questionnaires per month, number of potential participants providing consent to be included in the research database rate, and number of included participants per recruitment site.

Recruitment site	Recruitment method	Recruitment period (Months)	Total number of filled in online questionnaires (N)	Average number filled in online questionnaire per month (N)	Number of women providing consent to be included in the research database (N, %)	Pilot trial participants (N, %)
Echo center 1	Questionnaire invitation sent^1^	19	1088	57	583 (54%)^4^	27 (68%)
Echo center 2	Questionnaire invitation sent^2^	12	441	37	212 (48%)^4^	12 (30%)
Lactational practice	Flyer in waiting room^3^	8	11	1	5 (45%)^4^	0 (0%)
Mental healthcare center 1	Flyer in waiting room^3^	8	3	0	3 (100%)^4^	1 (3%)
Mental healthcare center 2	Flyer in waiting room^3^	8	0	0	0 (0%)	0 (0%)
Academic hospital	Flyer in waiting room^3^	10	30	3	14 (48%)^4^	0 (0%)
Total		–	1573	–	817 (52%)	40 (3%)

^1^The online questionnaire invitation was sent to potential participants 3 weeks after the 20-week ultrasound.

^2^When women visited echo centre 2 for their 20-week ultrasound, personal email addresses were collected; 3 weeks thereafter the online questionnaire invitation was sent per email.

^3^Flyer with link to online questionnaire was located in the waiting room.

^4^Percentage of total number of filled in online questionnaires per recruitment site.

Regarding reasons for (non) participation, there were four specific moments when participants made the decision to join the study: *after speaking with researcher by phone*, *after speaking to midwife*, *after speaking to partner*, and *after reading study information*. For some participants, there was *no specific moment* when they made the decision to join (see Table 4). Four main reasons concerning reasons why participants participated in the pilot trial were identified: *potential benefits, study topic, familiarity with research,* and *moral obligation.* The most widely reported reason to join the study was the potential benefit that participation might bring participants, and that participation might help other women, healthcare and science (see Table 5).

**Table 4:** Description of reported moments of decision to participate in the pilot trial.

Moment	Specific elements	Representative comments from participants
1. After speaking with researcher by phone	Personal interaction with the researcher, personal approach	“After filling in the online questionnaire, I was positive about joining a study, and when I heard you on the phone I decided to join”
	Safety reassurance	“I decided to join the study after I was able to ask you my remaining questions regarding safety”
	Discussing protocol compliance	“When I heard that I could leave out the collection of some samples, if it would be too much of a burden for me”
2. After speaking to midwife	Midwife’s reassurance and encouragement	“After I spoke with you on the phone, I discussed the study with my husband and midwife. The fact that my midwife said that she had also thought about advising me to sign up was especially reassuring.”
3. After speaking to partner	Partner’s agreement and encouragement	“After discussing the study with my husband; he was very enthusiastic about the study”
4. After reading study information		“After reading the study information, I directly emailed and signed up”
5. No specific moment		“There was no specific moment. I first had to let the information sink in, because at first I was afraid that it would cost too much time”

**Table 5:** Description of reasons for participation in the pilot trial.

Reason	Specific elements	Representative comments from participants
1. Potential benefits	Personal yield	“In my situation it is appealing to me to receive something that might help me, even if it is a placebo effect”
	Potential benefit for other people	“I’m joining the study because it eventually might help other people to reduce or prevent symptoms”
	Potential benefit for healthcare	“I had depressive symptoms after my first pregnancy. It would be nice if there was an alternative to medication to reduce depressive symptoms”
	Potential benefit for science	“I believe it is important to help scientific research, and with that the health of mothers and babies”
	Cost/benefit analysis	“I like to contribute to studies which pose no burden on my child and which contribute to something I find useful. Given that your study included both, I directly signed up to join. To me, research is useful when it is more focused on healthy life style and eating patterns than on medication”
2. Study topic	Personal interest in (gut) microbiota, mind–body interaction, diet, mood	“I choose to join because the research topic appeals to me: the link between the brain/mood/mental wellbeing, the gut and diet”
	Prenatal anxiety and depression being a taboo subject	“Prenatal anxiety and depression are important themes, but they’re a taboo”
	Recognizing oneself in the study population	“I am familiar with having depressive symptoms during pregnancy”
	Through friends/family being familiar with food supplements, or anxiety and depression	“I have a brother who mentally felt much better after taking vitamin D (..) and a sister-in-law who was suicidal during her pregnancy”
3. Familiarity with research	Probiotic product Previous participation in research	“I’m joining because this study includes a safe and natural product (I would not have participated if a medication would have been tested)” “In the past, I joined a scientific research study on gut microbiota”
4. Moral obligation	Important to participate in scientific research to generate more knowledge	“I see it as a moral obligation to join: this research is important and therefore important to contribute to it”

**Figure 2:**
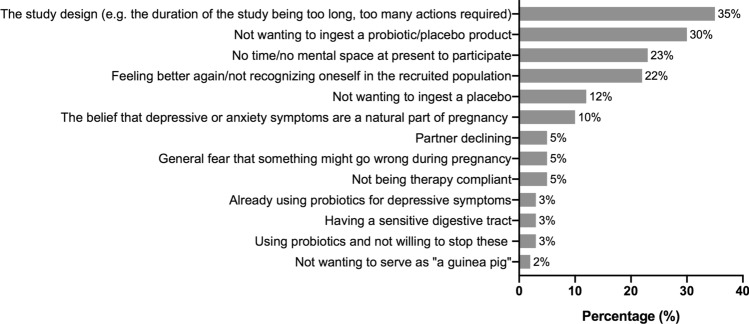
Reasons for declining study participation as a percentage of the total number of women who were invited to join the pilot trial and provided reasons for declining participation (N = 60).

Sixty women provided reasons why they did not want to participate after receiving study information. The main reason to decline participation was the study design (e.g. the duration of the study being too long, too many actions required) (Fig. 2).

#### Retention

Ninety-eight percent of recruited participants completed the pilot trial. One participant was unreachable to schedule the closing visit (see Fig. 1).

#### Compliance

Thirty-nine participants (98%) complied with the intervention regime of > 80% of probiotic and placebo intake during the first 8 weeks. For one participant, compliance was unknown during the first eight weeks because of the participant being unreachable to schedule a home visit. Thirty-six women (90% of the total population) adhered to probiotic or placebo ingestion of > 80% of probiotic intake between t1 up to delivery. Several reasons were reported for non-adherence between t1 and delivery: daily intake posed too much burden on daily life (*n* = 1), not observing an effect on mood (*n* = 1), receiving new medication and not wanting to ingest the sachets and medication in parallel (*n* = 1). One participant was unreachable at the end of the study. Furthermore, the compliance criterion for the evaluation form was met with 98% of women filling in the evaluation form. Other compliance criteria that were not met included: filling in the cry diary and collecting vaginal and microbial samples (83–85% compliance rate) (see Table 1). Participants who did not collect these data indicated that it posed too much burden on their daily life to fill in the cry diary and/or to collect biological samples.

#### Participants’ experiences

The progression criterion for level of acceptability of the pilot in principle was met (i.e., likelihood of future participation in a trial; mean score of 8, *SD* = 1.7; scale range = 1–10), but not the acceptability of the pilot in practice (mean score of 4, *SD* = 2.2; scale range = 1–10, lower score indicating lower perceived burden) (see Table 1). Regarding the level of acceptability of electronic questionnaires and collection of biological samples, the majority of participants indicated that they found the number of online questionnaires during the pilot trial appropriate (95%) and the online method of filling in questionnaires convenient (87%). With respect to the collection of maternal stool and vaginal samples, most women indicated that clear user guides were provided (88% and 91%, respectively) and that the number of samples to be collected was doable/appropriate (88% and 76%, respectively). Regarding the collection of infant stool samples, 33% of women indicated that the collection guide was clear and 30% found the number of infant samples to be collected appropriate. Most women indicated that they had no problem with collection of hair (88%), however some women (9%) found it a little scary to have hair collected (see Supplementary Fig. 2A–E). Most positive experienced aspects of the pilot trial were the personal guidance and interaction with the researcher (37%) and the home visits (24%). Most negative experienced aspects were the collection of biological samples (32%) and the ingestion of the probiotic/placebo product (24%) (see Supplementary Fig. 3A,B).

**Figure 3:**
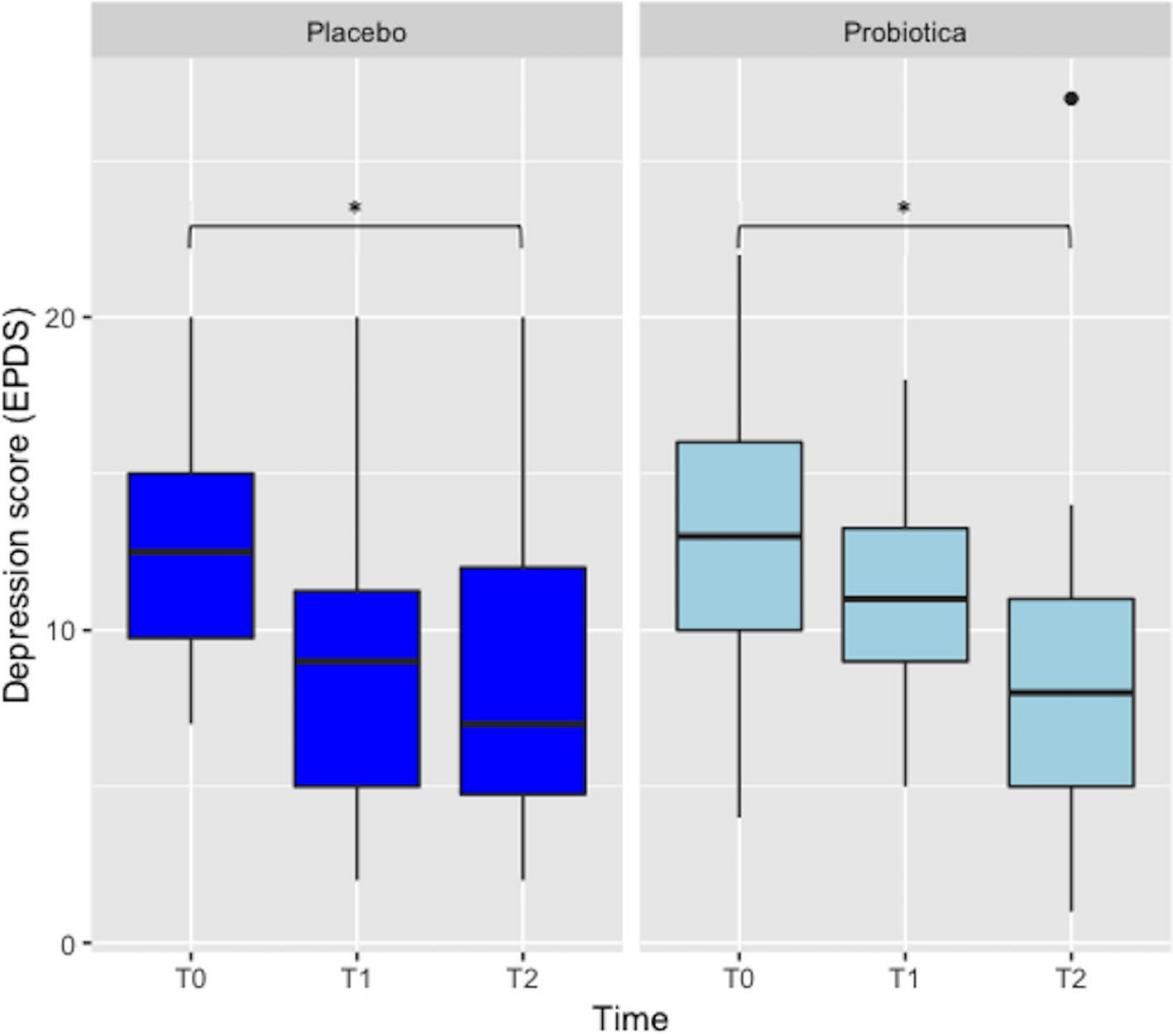
Depressive symptom scores of women in the probiotic (N = 20) and placebo groups (N = 20) measured at baseline (t0), 8 weeks after probiotic/placebo intake (t1) and 4 weeks postpartum (t2). *p < 0.05.

### Secondary outcomes

Maternal psychosocial distress (i.e., depressive symptoms, anxiety, stress) was scored both pre-treatment (t0; *M* = 27.8 weeks gestation, *SD* = 1.5), after 8 weeks of probiotic/placebo intake (t1; *M* = 35.8 weeks gestation, *SD* = 1.5) and 4 weeks post-partum (t2; *M* = 4.35 weeks postpartum, *SD* = 0.8). Note that depression/cognitive reactivity to sad mood (LEIDS-R)*,* pregnancy-related anxiety (PRAQ-R), pregnancy-related hassles (PES), general daily hassles (APL), and antenatal attachment (MAAS) questionnaires were only filled in at t0 (*M* = 27.8 weeks gestation, *SD* = 1.5) and t1 (*M* = 35.8 weeks gestation, *SD* = 1.5). The Maternal Postnatal Attachment Scale (MPAS) was filled in at t2 (*M* = 4.35 weeks postpartum, *SD* = 0.8). For the secondary analyses, all 40 participants were included in the originally assigned groups.

#### Depression (EPDS)

ANOVAs performed on the EPDS score revealed a main negative effect of time [F(2,76) = 11.2, *p* < 0.05]. No effect was observed for group [F(2,38) = 0.8, *p* > 0.05] nor for a time by group interaction [F(2.76) = 0.84, *p* > 0.05]. Tukey HSD *post-hoc* tests were performed and revealed that participants in the probiotic group had a significant decrease in depressive symptoms between baseline (t0) and 4 weeks postpartum (t2) (*p* = 0.04). Similarly, depressive symptoms of women in the placebo group significantly decreased between baseline (t0) and 4 weeks postpartum (t2) (*p* = 0.04). Thus, depressive symptoms decreased in the group as a whole from baseline to after the intervention, irrespective of study group (see Fig. 3).

#### Depression/cognitive reactivity to sad mood (LEIDS-R)

ANOVAs on LEIDS-R total score showed a significant negative effect of time [F(1,38) = 12.55, *p* = 0.02] and group [F(1,38) = 5.82, *p* < 0.001], but no time by group interaction [F(1,38) = 0.92, *p* > 0.05]. Tukey HSD *post-hoc* test showed that women in the placebo group had significantly lower total scores on cognitive reactivity at t0 than women in the probiotic group at t1 (*p* = 0.001).

#### General anxiety (STAI-S)

**Figure 4:**
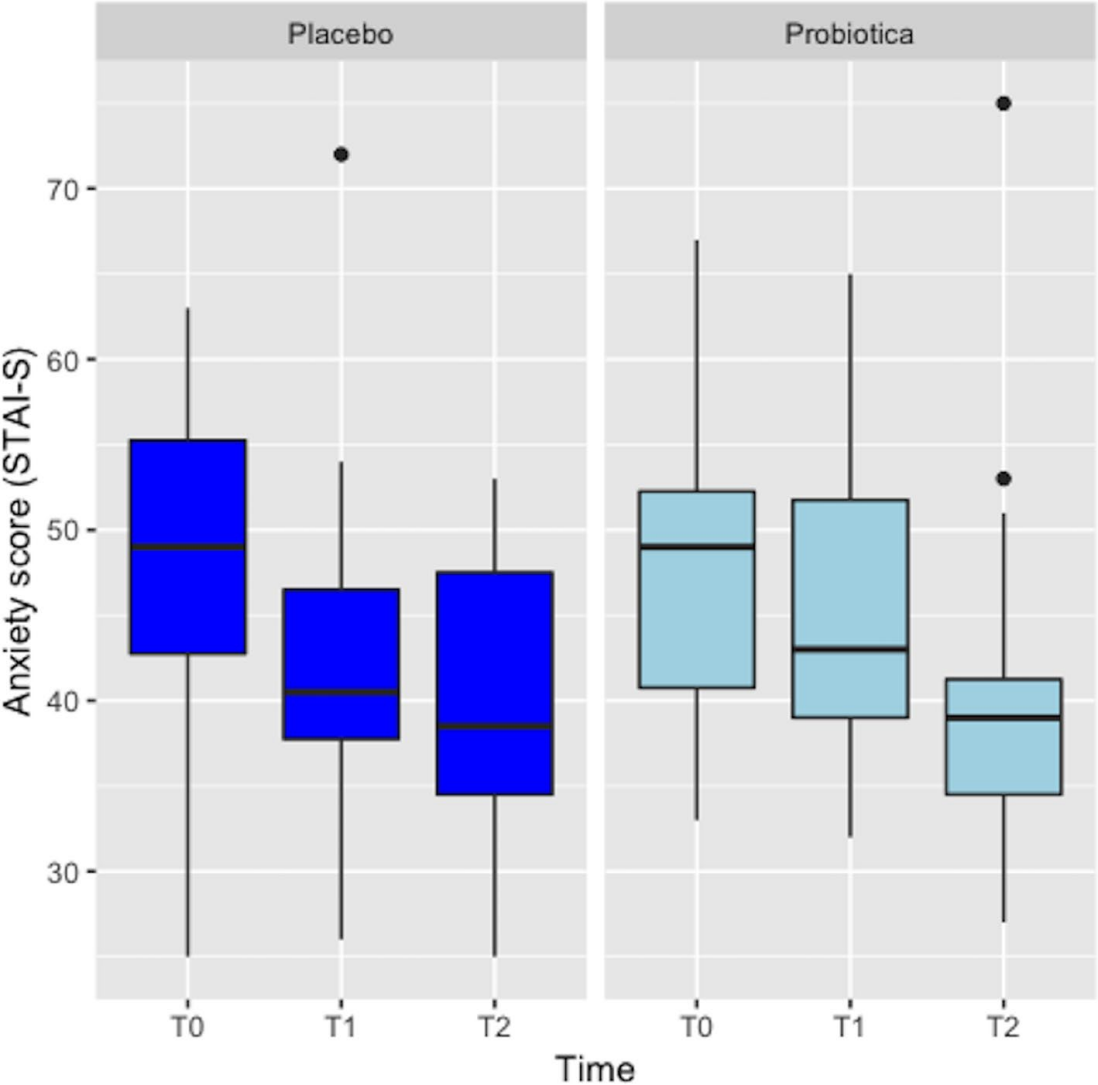
Anxiety symptom scores of women in the probiotic (N = 20) and placebo groups (N = 20) measured at baseline (t0), 8 weeks after probiotic/placebo intake (t1) and 4 weeks postpartum (t2).

Symptoms of general anxiety decreased in both groups. ANOVAs revealed a significant negative effect of time on general anxiety [F(1,38) = 10.79, *p* < 0.05], but not for group [F(1,38) = 0.34, *p* > 0.05] and time by group interaction [F(1,38) = 0.47, *p* > 0.05]. Tukey HSD *post-hoc* test revealed that the decrease in anxiety was only significant for the total population (*p* < 0.001) (Fig. 4).

#### Pregnancy-related anxiety (PRAQ-R)

A significant negative effect of time on pregnancy-related anxiety was found for the subscale fear of bearing a handicapped child [F(1,38) = 6.37, *p* < 0.05], but no effect was observed for time by group interaction [F(1,38) = 0.7, *p* > 0.05]. For the subscale fear of giving birth, no significant effects of time [F(1,38) = 2.5, *p* > 0.05], group [F(1,38) = 0.2, *p* > 0.05], nor time by group interaction were found [F(1,38) = 0.28, *p* > 0.05].

#### Pregnancy-related daily hassles and general daily hassles (PES and APL)

There was a significant negative effect of time on pregnancy-related daily hassles (PES composite ratio score; [F(1,38) = 14.6, *p* < 0.05]) and general daily hassles (APL stress score; [F(1,38) = 13.9, *p* < 0.05]). Tukey HSD *post-hoc* showed no significant decrease in pregnancy-related daily hassles from baseline to t1 in the probiotic group (*p* = 0.18) or placebo group (*p* = 0.19). Similarly, the decrease in general daily hassles was not significant in neither probiotic nor placebo group (*p* = 0.37, *p* = 0.38, respectively).

#### Maternal Antenatal Attachment Scale (MAAS)

ANOVAs yielded a main positive effect for time on maternal attachment score, with maternal attachment increasing for the group as a whole [F(1,38) = 27.8, *p* < 0.05]. Tukey *post-hoc* test showed no significant time by group interaction (*p* = 1).

#### Maternal Postnatal Attachment Scale (MPAS)

Women in the probiotic group (*M* = 67.35, *SD* = 8.42) and women in the placebo group (*M* = 68.85, *SD* = 7.08) had comparable attachment scores postpartum (*t*^38^ = 0.6, *p* = 0.55).

#### Side effects and serious adverse events

No significant differences were found in the number of side effects and SAEs between the probiotic and placebo groups (Fisher’s exact test, *p* = 0.16, *p* = 1, respectively). Seventeen participants in the probiotic group and 12 participants in the placebo group reported side effects during intake of the probiotic/placebo product. Most reported side effects were constipation (10 probiotic group vs. 5 placebo group), flatulence (6 probiotic group vs. 9 placebo group), nausea (7 probiotic group vs. 4 placebo group), and diarrhea (5 probiotic group vs. 3 placebo group) during the first 8 weeks of probiotic intake. Four SAEs occurred: in the probiotic group, two participants were admitted to the hospital, one for pregnancy induced hypertension and the second for preterm delivery (34 weeks gestational age). In the placebo group, one participant was admitted due to severe headache and another participant had placenta praevia. No SAEs were related to study participation.

## Discussion

The PIP pilot trial was generally feasible and acceptable. Women were positive about future participation in a similar trial, dropout rate was minimal, and compliance to the study protocol high. However, the recruitment and acceptability of the trial in practice criteria (i.e., the perceived burden of the study protocol) were not met. Secondary data analyses revealed a decrease in pregnancy psychosocial distress, but there was no significant difference between the probiotic and placebo groups for these outcomes. Similarly, there were no significant differences between the probiotic and placebo groups for the long-term outcomes.

While retention was very high with minimal dropout, recruitment was problematic in this study. Of the 1573 women screened, 3% were randomized. This randomization rate is similar to the Milgrom et al. (2011) study on the effectiveness of a behavioral intervention for reducing depressive symptoms among pregnant women^49^. However, the participation rate of this study (30%) was lower than the Milgrom et al. (2011) study participation rate (56%). While women in the Milgrom et al. (2011) study were screened and offered participation at clinical centers by midwives, recruitment in the current study was conducted through telephone invitation by a researcher, possibly explaining the difference in participation rates between the studies. Clinical staff are indeed known good partners to facilitate recruitment by identifying and speaking directly to eligible pregnant women about study protocols^50^. In light of our results, these approaches might be even more essential for pregnant women with psychosocial distress. Future studies investigating food supplements in this population may hence consider having midwives or mental healthcare professionals personally introduce the trial to pregnant women. In addition, to ensure constant and effective recruitment, a future full trial should invest in strong collaboration between researchers, midwives, and mental healthcare professionals^51^. Additionally, the results of our qualitative analyses showed that the main reason of women to decline study participation was the burden of the study. One method to reduce the burden of the study and improve recruitment rates would be meeting with women after regular pregnancy care visits and guiding and supporting them through the study’s various steps. Another would be to shorten the duration of the study by limiting it to pregnancy (i.e. leaving out child outcome measures). Finally, for a larger trial focused on clinical outcomes, the collection of biological samples could be left out. Alternatively, recruitment staff should invest more time in better explaining the value of collecting biological samples to potential participants.

Although measured in a small population, this study found no effect of probiotics on psychosocial distress after 8 weeks of probiotic intake. Note that in our study we investigated group effects of probiotics, while it is known that effects can vary from one individual to another. Factors that can influence probiotic effects are host diet^52^, genetics, age, medication use^53^, severity of symptoms (e.g. mild, moderate, severe depression)^9^, and gut microbiota composition^54^. In future studies, these factors could be included to assess whether they would predict who would benefit from probiotic supplementation. For instance, baseline gut bacterial samples can be collected and bacterial profiles used as predictors of probiotic response^55^.

The lack of effect of 8 weeks of probiotic intake on psychosocial distress is in contrast with the Slykerman et al. (2017) study, which reported a significant positive effect of perinatal maternal probiotic intake on postpartum depressive and anxiety symptoms^10^. The difference in outcomes may be explained by methodological differences between the studies. The Slykerman et al. (2017) study included a substantially larger sample than the current study (*n* = 423) and used *Lactobacillus rhamnosus* HN001, whereas our sample size was relatively small (*n* = 40) and participants received a probiotic mixture without this strain. A recent meta-analysis indicated that probiotic effects are strain and species-specific^56^. Furthermore, differences in the timing and length of probiotic intake may, in part, explain the differences in outcomes^57^.

The general reduction of psychosocial distress after 8 weeks of probiotic intake may indicate a natural decrease of symptoms. Studies report that anxiety (i.e., state and trait) naturally decreases over gestation^58–60^ and spontaneous improvement of symptoms often occurs in RCTs that include participants with high depression symptom scores^61^. However, it is possible that two other factors played a role in reduction of symptoms: participants’ expectancy to respond to the intervention product^62–64^ and strong engagement between research staff and participants^65,66^. In support of the former, the qualitative results of this study showed that most women indicated that their main reason for participation was the potential benefit it might bring them. Regarding research staff and participant engagement, a recent RCT investigating the same probiotic product also reported a reduction in depressive symptoms after 8 weeks of probiotic and placebo intake, but no significant difference between the probiotic and placebo groups. Similar to the current study, research staff was in close contact with participants throughout the study^65^. Thus, at this point, it is unclear whether the decrease in symptoms after 8 weeks of product intake was a natural phenomenon that resulted from participants’ expectancy to respond to the intervention product, was due to strong engagement between with the research staff, or a combination of all factors. Future RCTs with blinded assessment should account for the possible effects of these factors on psychosocial distress by including a third control arm receiving no intervention.

The strength of this pilot study is the mixed methods approach of combining both qualitative and quantitative measures. This method provided an in depth portrayal of reasons for joining and declining study participation in women with psychosocial distress. Other strengths of the study are the inclusion of confounders and the high compliance rate of probiotic/placebo intake and mood questionnaires, which provided a realistic and unbiased preliminary result of the probiotic/placebo intervention for the secondary outcomes.

Several limitations should be mentioned regarding the study results. First, the study included a relatively small sample size to explore potential efficacy of a probiotic product, and therefore these secondary outcomes should be interpreted with caution. Second, given that this study did not include a third control arm, the possibility of a natural decrease of symptoms should be acknowledged. Third, the study population included a relatively large number of women of higher socio-economic status, which hampers the generalizability of the study results. Finally, the study included a relatively large number of women with subclinical depressive and anxiety symptoms (i.e. without a diagnosis of depression or anxiety disorder). Previous systematic reviews and meta-analyses have shown that lower depressive symptom severity is positively associated with a higher placebo response, and thus it is possible that the placebo response was relatively high in the current pilot trial^67–69^.

The PIP pilot trial was generally feasible and acceptable, but suffered from low recruitment rates. Alternative methods of recruitment, including active recruitment by healthcare professionals, might be considered in a future definitive trial. Although evaluated in a relatively small population, analyses of secondary endpoints did not reveal differences between probiotic and placebo groups. In light of the increased risk of adverse maternal and infant outcomes for women suffering from prenatal depression and anxiety, future studies should explore the potential efficacy of other study designs or other (non) pharmacological treatments. Possibilities are, for example, to reduce the number of study measures and to use the same probiotic supplement starting in the first trimester of pregnancy, when natural pregnancy changes to the maternal gut microbiota have not yet taken place^70^ and the probiotic may have a larger effect. Alternatively, the use of other probiotics or combinations of probiotics and dietary fibers that promote the activity of the probiotic bacteria should be explored^71,72^.

## Supplementary Information

Supplementary Information.

## Data Availability

The datasets generated during and analyzed during the current study are available from the corresponding author on reasonable request.

## Abbreviations

APL: Dutch Everyday Problem List
CRF: Case Report Form
EPDS: Edinburgh Postnatal Depression Scale
CI: Coordinating investigator
LEIDS-R: Leiden Index of Depression Sensitivity-Revised
MAAS: Maternal Antenatal Attachment Scale
ETC: Medical Ethics Committee
MPAS: Maternal Postnatal Attachment Scale
PSQI: Pittsburgh Sleep Quality Index
PES: Pregnancy Experience Scale
PRAQ-R: Pregnancy-Related Anxiety Questionnaire-Revised
RCTs: Randomized clinical trials
STAI-S: State-trait Anxiety Inventory
AEs: Serious Adverse Events
