# Supplementary material for: Probiotics as a treatment for prenatal maternal anxiety and depression: a double-blind randomized pilot trial
Source: Sci Rep. 2021 Feb 4;11:3051. doi: 10.1038/s41598-021-81204-9 (PMC7862351; doi:10.1038/s41598-021-81204-9)
Supplement: Supplementary file 1 — Supplementary Information. [file 41598_2021_81204_MOESM1_ESM.pdf]

Probiotics as a treatment for prenatal maternal anxiety and depression: a double-blind randomized pilot trial

Pamela D. Browne[1,2*], Antoinette C. Bolte[3], Isolde Besseling-van der Vaart[4], Eric Claassen[2], Carolina de Weerth[1]

Supplementary Figure 1 Self-created stressful events questionnaire.

Indicate whether each of the following events occured in the past year (yes/no):

1) Serious illness (yourself)
2) Serious illness (your partner)
3) Death of a close family member of friend
4) Divorce/break up with your partner
5) Relational/marital problems
1) Financial problems
2) Domestic violence
3) Other major or stressful events, namely ….

Supplementary Figures 2A-E. Participants' responses as a percentage of the total women reporting on their experiences with filling in online questionnaires (N=38), collecting stool samples (N=34) and vaginal samples (N=33), and having hair collected during the study period (N=34). Grey bars indicate positive experiences; dotted bars indicate negative experiences.

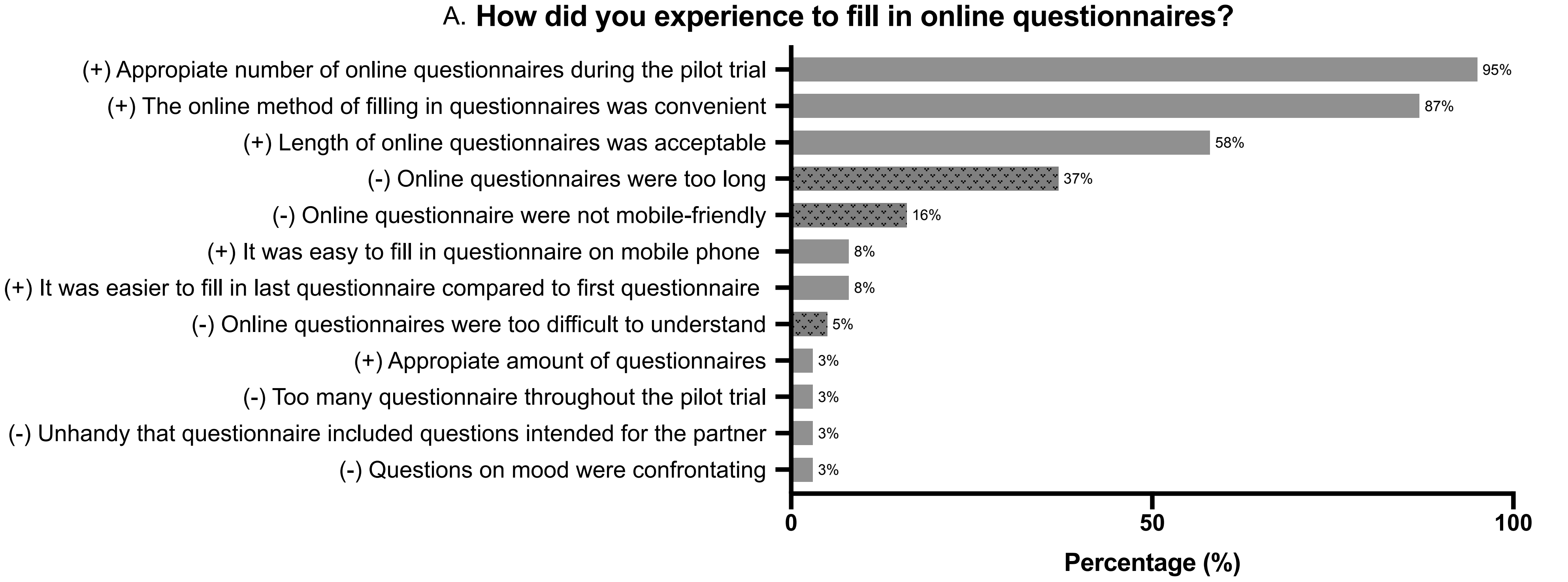

A. How did you experience to fill in online questionnaires?
(+) Appropiate number of online questionnaires during the pilot trial
95%
(+) The online method of filling in questionnaires was convenient
87%
(+) Length of online questionnaires was acceptable
58%
(-) Online questionnaires were too long
37%
(-) Online questionnaire were not mobile-friendly
16%
(+) It was easy to fill in questionnaire on mobile phone
8%
(+) It was easier to fill in last questionnaire compared to first questionnaire
8%
(-) Online questionnaires were too difficult to understand
5%
(+) Appropiate amount of questionnaires
3%
(-) Too many questionnaire throughout the pilot trial
3%
(-) Unhandy that questionnaire included questions intended for the partner
3%
(-) Questions on mood were confrontating
3%
0
50
100
Percentage (%)

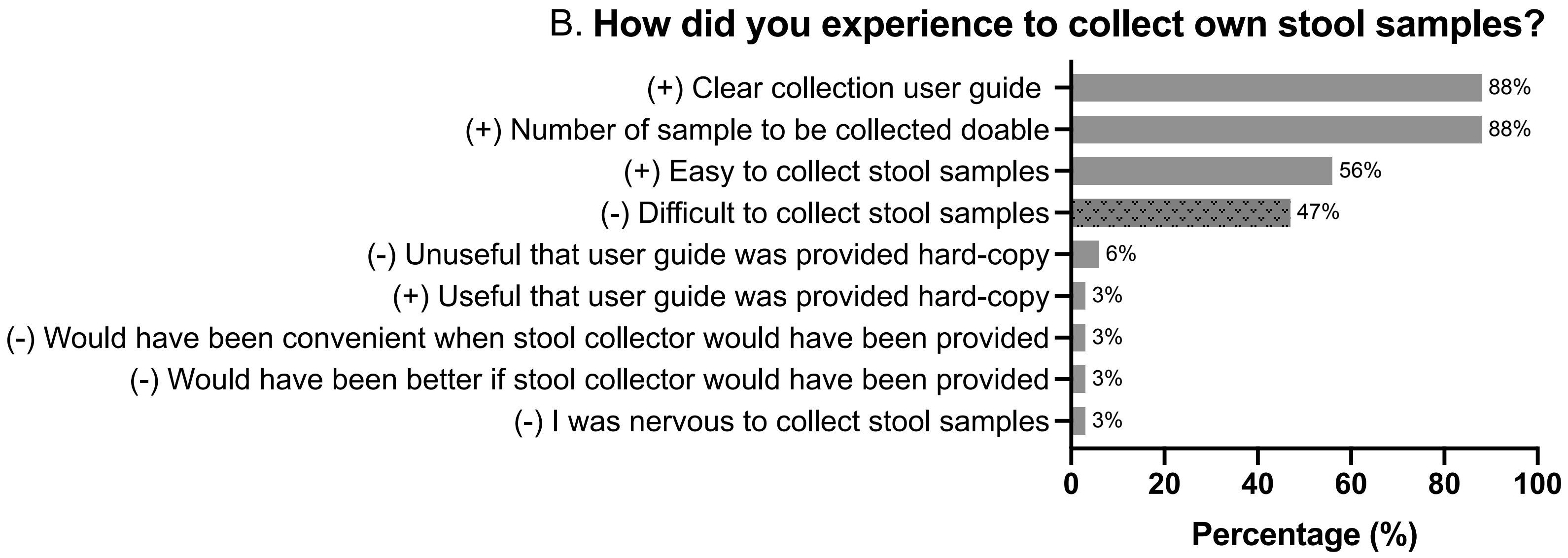

B. How did you experience to collect own stool samples?
(+) Clear collection user guide
(+) Number of sample to be collected doable
(+) Easy to collect stool samples
(-) Difficult to collect stool samples
(-) Unuseful that user guide was provided hard-copy
(+) Useful that user guide was provided hard-copy
(-) Would have been convenient when stool collector would have been provided
(-) Would have been better if stool collector would have been provided
(-) I was nervous to collect stool samples
88%
88%
56%
47%
6%
3%
3%
3%
3%
0
20
40
60
80
100
Percentage (%)

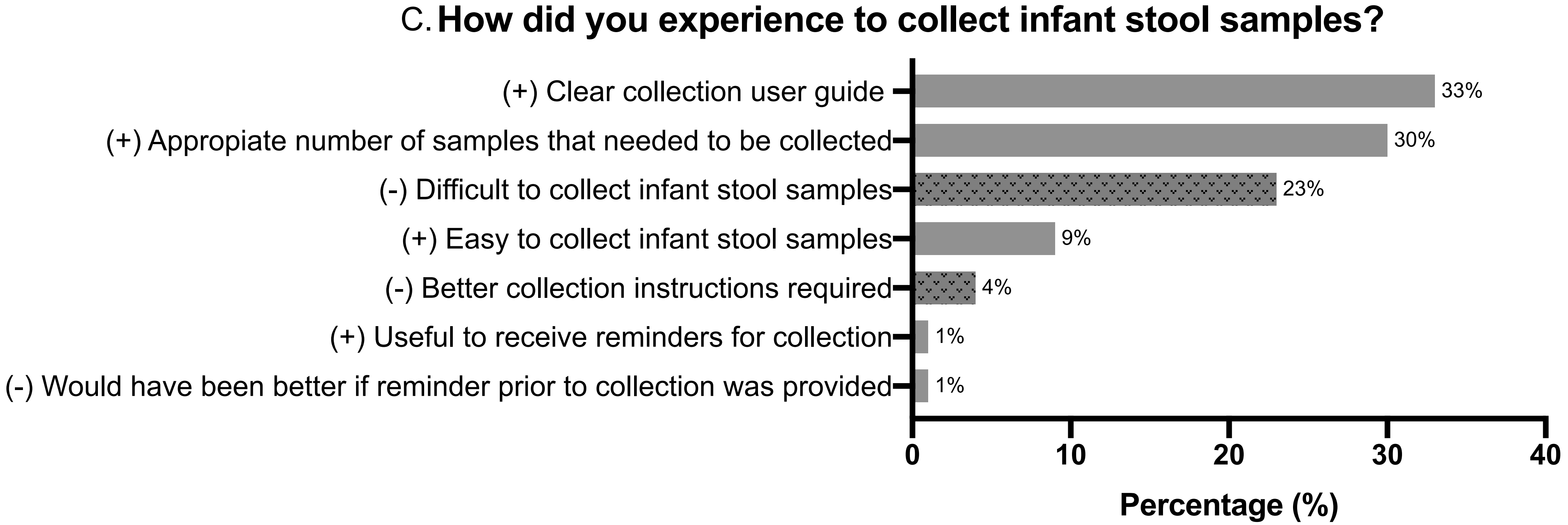
C. How did you experience to collect infant stool samples?
(+) Clear collection user guide
(+) Appropiate number of samples that needed to be collected
(-) Difficult to collect infant stool samples
(+) Easy to collect infant stool samples
(-) Better collection instructions required
(+) Useful to receive reminders for collection
(-) Would have been better if reminder prior to collection was provided
33%
30%
23%
9%
4%
1%
1%
0
10
20
30
40
Percentage (%)

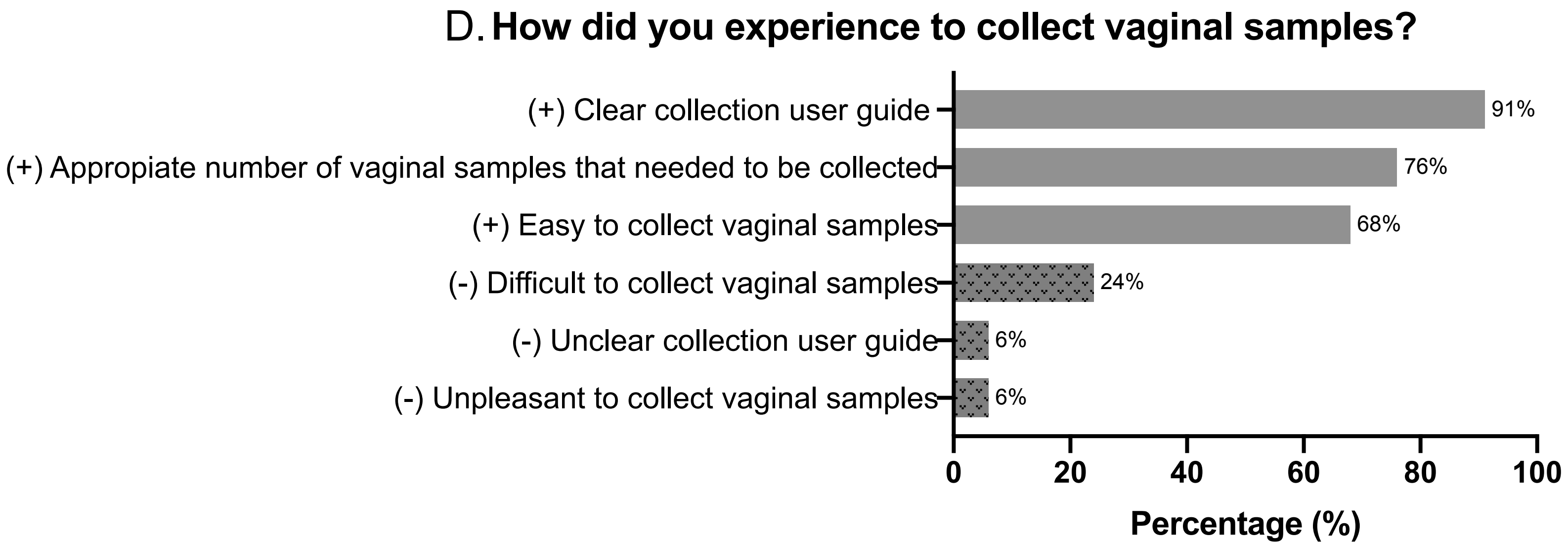
D. How did you experience to collect vaginal samples?
(+) Clear collection user guide
(+) Appropiate number of vaginal samples that needed to be collected
(+) Easy to collect vaginal samples
(-) Difficult to collect vaginal samples
(-) Unclear collection user guide
(-) Unpleasant to collect vaginal samples
91%
76%
68%
24%
6%
6%
0
20
40
60
80
100
Percentage (%)

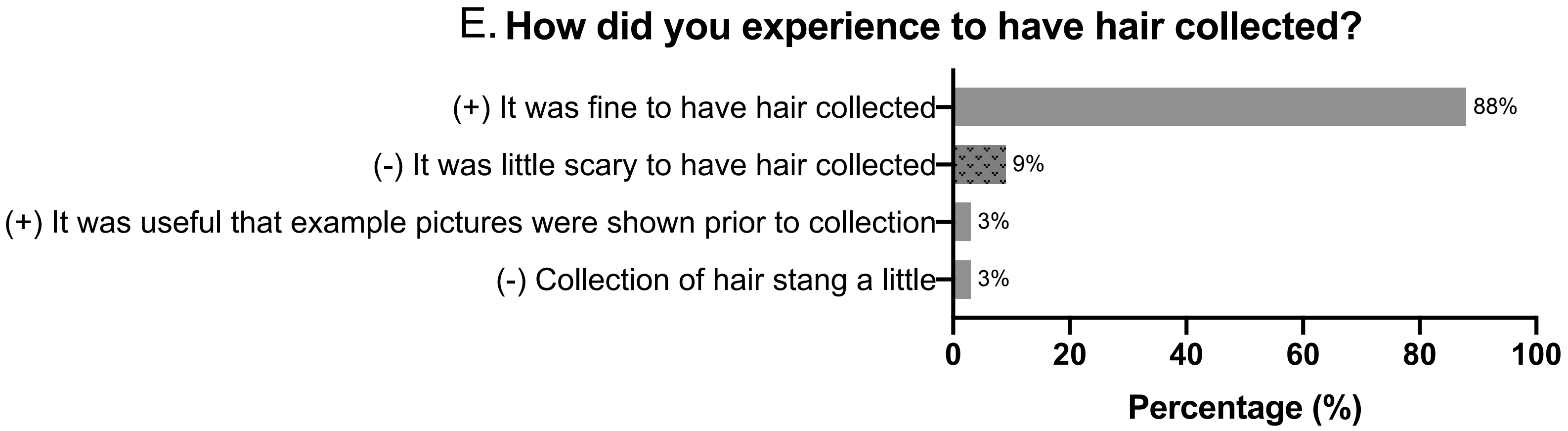
E.
How did you experience to have hair collected?
(+) It was fine to have hair collected
(-) It was little scary to have hair collected
(+) It was useful that example pictures were shown prior to collection
(-) Collection of hair stang a little
88%
9%
3%
3%
0
20
40
60
80
100
Percentage (%)

Supplementary Figures 3A-B. Participants' reported positive and negative aspects of the pilot trial as a percentage of the total women filling in the evaluation form (N=38).

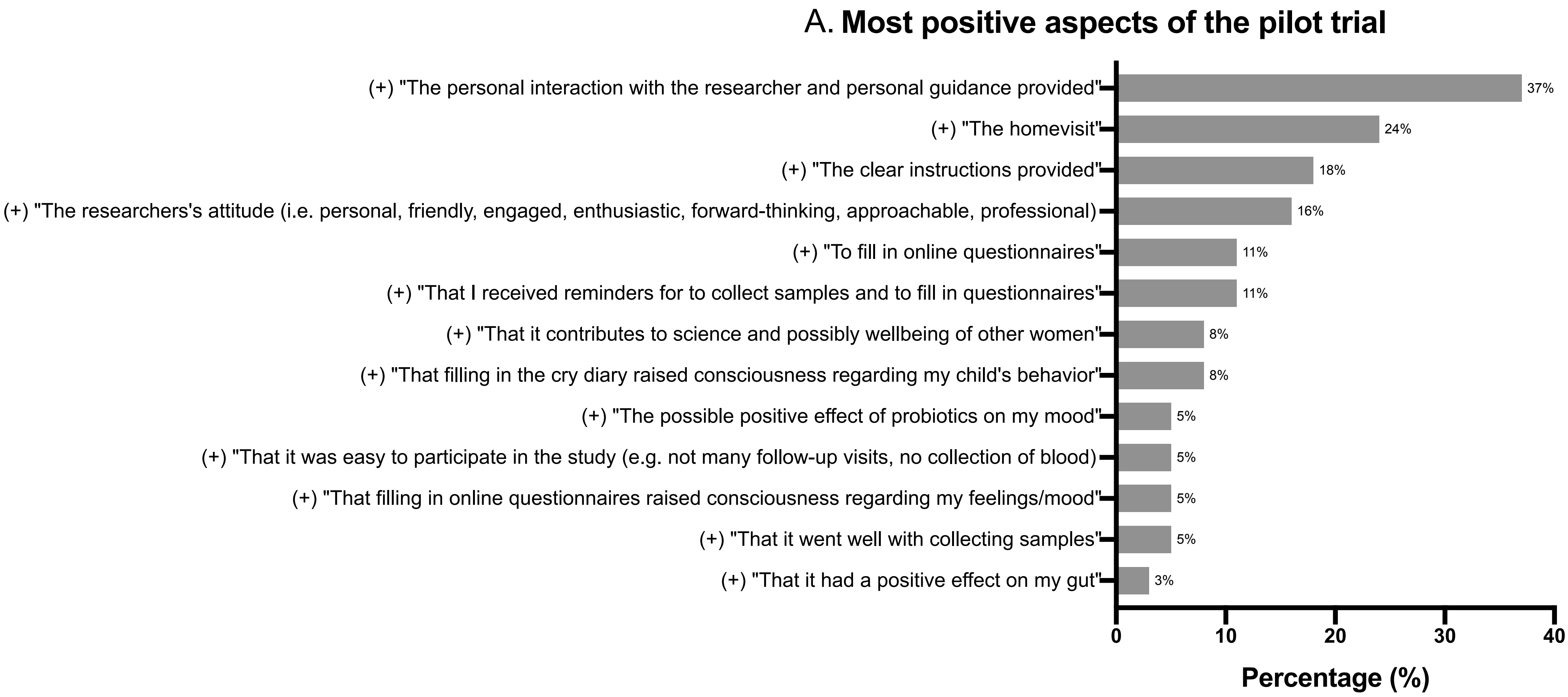
A. Most positive aspects of the pilot trial
(+) "The personal interaction with the researcher and personal guidance provided"
(+) "The homevisit"
(+) "The clear instructions provided"
(+) "The researchers's attitude (i.e. personal, friendly, engaged, enthusiastic, forward-thinking, approachable, professional)
(+) "To fill in online questionnaires"
(+) "That I received reminders for to collect samples and to fill in questionnaires"
(+) "That it contributes to science and possibly wellbeing of other women"
(+) "That filling in the cry diary raised consciousness regarding my child's behavior"
(+) "The possible positive effect of probiotics on my mood"
(+) "That it was easy to participate in the study (e.g. not many follow-up visits, no collection of blood)
(+) "That filling in online questionnaires raised consciousness regarding my feelings/mood"
(+) "That it went well with collecting samples"
(+) "That it had a positive effect on my gut"
37%
24%
18%
16%
11%
11%
8%
8%
5%
5%
5%
5%
3%
0
10
20
30
40
Percentage (%)

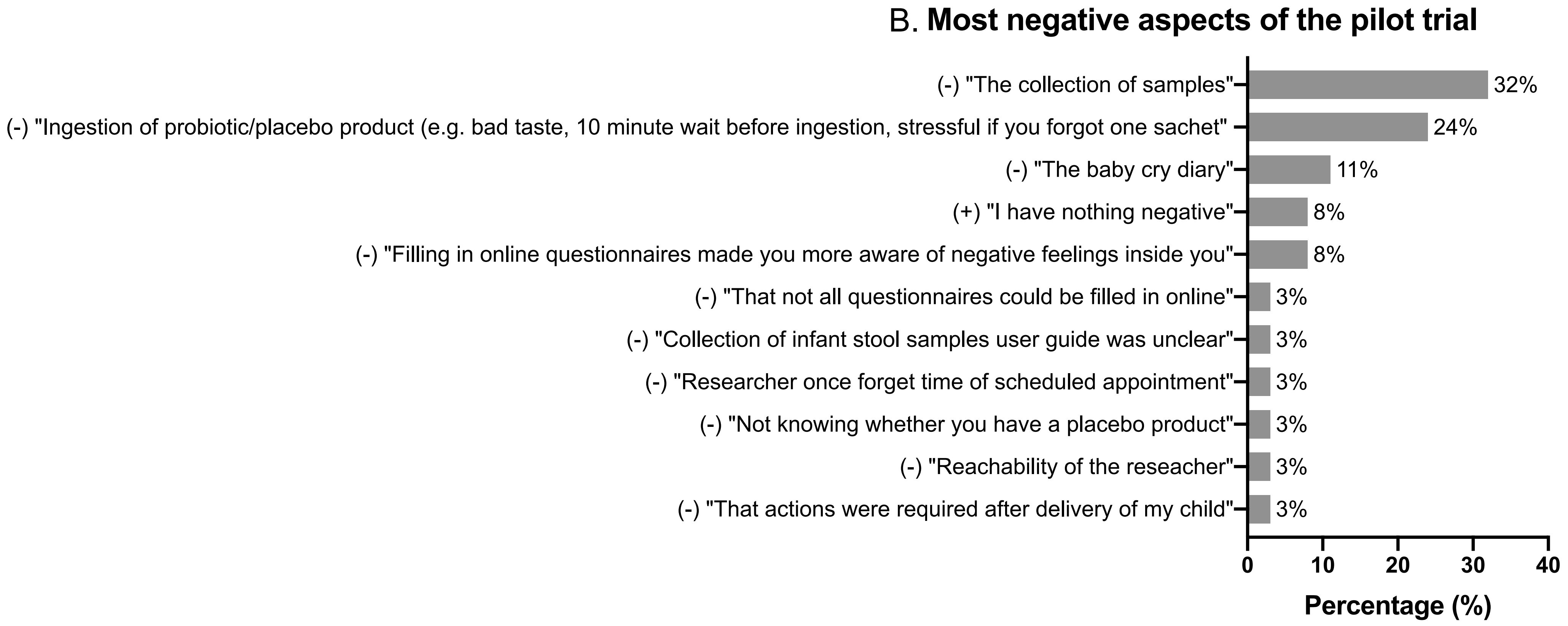

B. Most negative aspects of the pilot trial
(-) "The collection of samples"
32%
(-) "Ingestion of probiotic/placebo product (e.g. bad taste, 10 minute wait before ingestion, stressful if you forgot one sachet"
24%
(-) "The baby cry diary"
11%
(+) "I have nothing negative"
8%
(-) "Filling in online questionnaires made you more aware of negative feelings inside you"
8%
(-) "That not all questionnaires could be filled in online"
3%
(-) "Collection of infant stool samples user guide was unclear"
3%
(-) "Researcher once forget time of scheduled appointment"
3%
(-) "Not knowing whether you have a placebo product"
3%
(-) "Reachability of the reseacher"
3%
(-) "That actions were required after delivery of my child"
3%
0
10
20
30
40
Percentage (%)
